# Emerging trends in the agri-food sector: Digitalisation and shift to plant-based diets

**DOI:** 10.1016/j.crfs.2022.11.010

**Published:** 2022-11-17

**Authors:** Abdo Hassoun, Fatma Boukid, Antonella Pasqualone, Christopher J. Bryant, Guillermo García García, Carlos Parra-López, Sandeep Jagtap, Hana Trollman, Janna Cropotova, Francisco J. Barba

**Affiliations:** a Univ. Littoral Côte d’Opale, UMRt 1158 BioEcoAgro, USC ANSES, INRAe, Univ. Artois, Univ. Lille, Univ. Picardie Jules Verne, Univ. Liège, Junia, F-62200, Boulogne-sur-Mer, France; b Sustainable AgriFoodtech Innovation & Research (SAFIR), Arras, France; c ClonBio Group LTD, 6 Fitzwilliam Pl, Dublin, D02 XE61, Ireland; d Department of Soil, Plant and Food Sciences, University of Bari, Via Amendola, 165/A, 70126, Bari, Italy; e Department of Psychology, University of Bath, Bath, United Kingdom; f Department of Agrifood System Economics, Institute of Agricultural and Fisheries Research & Training (IFAPA), P.O. Box 2027, 18080, Granada, Spain; g Sustainable Manufacturing Systems Centre, School of Aerospace, Transport and Manufacturing, Cranfield University, Cranfield, MK43 0AL, United Kingdom; h Department of Work, Employment, Management and Organisations, School of Business, University of Leicester, Brookfield, 266 London Road, Leicester, LE2 1RQ, United Kingdom; i Department of Biological Sciences Ålesund, Norwegian University of Science and Technology, Larsgårdsvegen 4, 6025 Ålesund, Norway; j Nutrition and Food Science Area, Preventive Medicine and Public Health, Food Science, Toxicology and Forensic Medicine Department, Faculty of Pharmacy, Universitat de València, Avda. Vicent Andrés Estellés, s/n, 46100, Burjassot, València, Spain

**Keywords:** Digitalisation, Food industry 4.0, Precision agriculture, Smart food factory, Sustainability, Vegan, Vegetarian diets

## Abstract

Our planet is currently facing unprecedented interconnected environmental, societal, and economic dilemmas due to climate change, the outbreak of pandemics and wars, among others. These global challenges pose direct threats to food security and safety and clearly show the urgent need for innovative scientific solutions and technological approaches. Backed by the current alarming situation, many food-related trends have emerged in recent years in response to these global issues. This review looks at two megatrends in agriculture and the food industry; the shift to vegetable diets and the digital transformation in food production and consumption patterns. On one side, several innovative technologies and protein sources have been associated with more sustainable food systems and enhanced nutritional quality and safety. On the other side, many digital advanced technologies (e.g., artificial intelligence, big data, the Internet of Things, blockchain, and 3D printing) have been increasingly applied in smart farms and smart food factories to improve food system outcomes. Increasing adoption of vegetal innovations and harnessing Industry 4.0 technologies along the food supply chain have the potential to enable efficient digital and ecological transitions.

## Highlights

• Agri-food systems are currently under unprecedented challenges and undergoing profound changes.
• Digitalisation and the shift to vegetal diets are among the strong megatrends in the food sector.
• Industry 4.0 technologies are accelerating the development of plant-based innovations.
• AI, IoT, BD, blockchain, and robotics are increasingly leveraged in smart farming and smart food factories.
• Adoption of digital technologies and plant-based foods can enhance food sustainability.

## Graphical abstract

**Figure:**
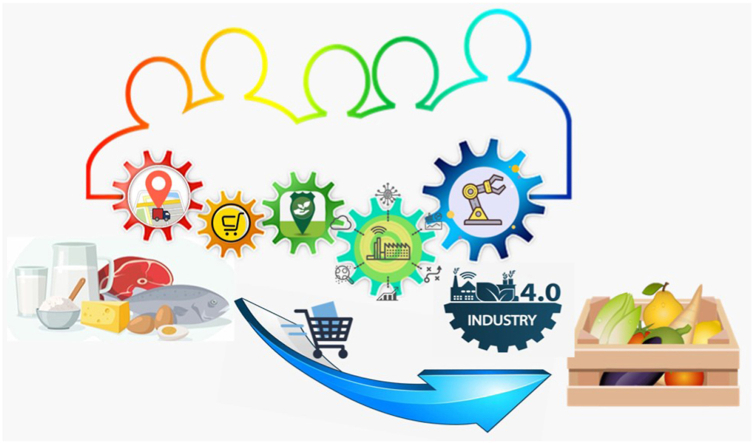

## 1 Introduction

Currently, the food supply chain is facing serious sustainability and security challenges that can be fuelled by climate change, the rapid growth in the world's population, and the ongoing international conflicts and pandemic outbreaks. Consequently, all actors within the food supply chain (e.g., manufacturers and retailers), as well as policy makers, and non-profit organizations are looking for innovative solutions to render the food system more sustainable and resilient. This has been backed by increasing consumers' awareness about the relatedness among their dietary habits, health, nutrition, and sustainability (Augustin and Cole, 2022; Giacalone et al., 2022; Zhang et al., 2022). Plenty of food trends have emerged or re-emerged in the last few years reaffirming the evolving attitude of consumers to rethink underpinning the convergence of health and the environment (Baker et al., 2022; Fiorentini et al., 2020; Hassoun et al., 2022a; Siegrist and Hartmann, 2020). These include, but are not limited to, the growing acceptance of novel or unusual food sources (e.g., insects, algae, food waste and by-products), food production systems (e.g., hydroponics, aquaponics, and aeroponics), and advanced technologies, such as cell-cultured, gene-edited, and 3D printed foods (Boukid et al., 2022; Bryant and Sanctorum, 2021; Hassoun et al., 2022b; Hubalek et al., 2022; Wood and Tavan, 2022).

Two megatrends, namely digitalisation and the shift to vegetal diets, have been increasingly looked for and are expected to increase even more in the coming years. Empowered by the fourth industrial revolution (Industry 4.0) advances, digital technologies have become more prevalent in different industries, including agriculture and the food industry. The key Industry 4.0 technologies have been widely reported with focus on the concept of smart farms and smart food factories. In the agri-food sector, the key Industry 4.0 technologies include among others artificial intelligence (AI), the Internet of Things (IoT), Big Data (BD), robotics, blockchain, 3D printing, and smart sensors (Hassoun et al., 2022c, 2022d). However, due to multiple obstacles, the shift to digital transformation is still at the early stage in the agri-food sector.

**Fig. 1:**
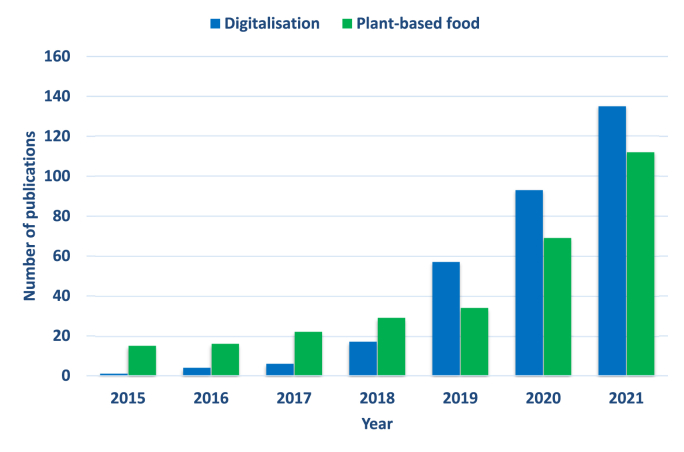
Evolution of the number of publications reporting on digitalisation and plant-based foods from 2015 to 2021. Data were obtained from Scopus in October 2022 with the following search criteria: For digitalisation; TITLE (digitalisation) OR (industry 4.0) OR (fourth industrial revolution) AND TITLE-ABS-KEY (food) OR (agriculture). For plant-based foods; TITLE (plant-based food).

In the frame of the transition towards plant-based diets, non-animal alternatives to meat, fish, egg and dairy are on the rise globally due to ethical, environmental, and health concerns. Nevertheless, the off-flavour of plant-based ingredients/foods, the lack of animal-like experience, and consumer attitude (e.g., meat lovers and neophobic eaters) are holding back this new segment to go mainstream (Bryant, 2022; Giacalone et al., 2022; Ishaq et al., 2022). For this reason, a lot of investments are flowing into plant-based research institutes and companies to find solutions. Even though intensive research is being conducted to address the limitations facing the digital/vegetal transitions (Fig. 1), to the authors’ knowledge this is the first time digital and vegetal transitions are discussed simultaneously to provide a full picture of the current situation and suggest future directions. Thus, the purpose of this review is i) to critically discuss current trends in plant-based foods and ii) to emphasize the role of Industry 4.0 technologies and digitalisation in boosting/consolidating the plant-based sector in the frame of shifting towards more sustainable food systems.

## 2 The trend of plant-based ingredients/foods

**Fig. 2:**
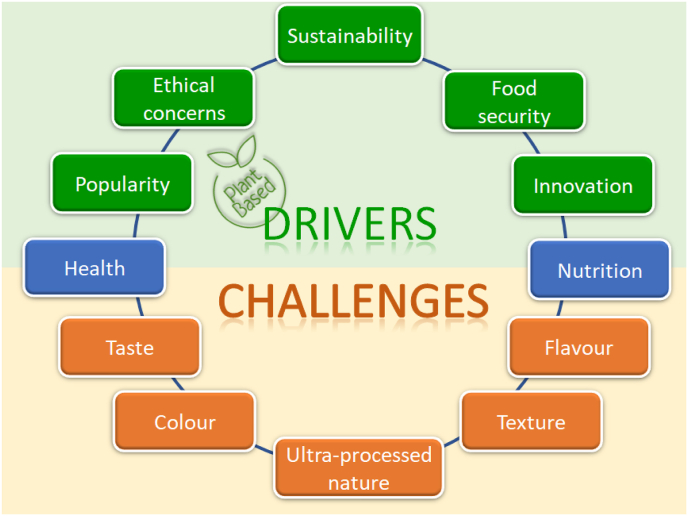
The main drivers and challenges of plant-based foods.

The trend to plant-based food has gained much popularity in recent years and it has been fuelled by many drivers including innovation, concerns about food security, and sustainability, among others. However, multiple challenges, especially consumer acceptance of sensory properties of plant-based ingredients/foods (e.g., flavour, texture, and colour) are still hindering them from reaching broader markets (Fig. 2).

### 2.1 Beyond vegan and vegetarian: flexitarian trend

Along with vegan and vegetarian trends, many consumers are following flexitarian diets by reducing their animal-derived foods to include more plant-based foods into their daily diets. Plant-based diets were reported to be health-beneficial due to reduced risk of cancer, obesity, and cardiovascular diseases (Craig et al., 2021; Samtiya et al., 2021). Public health guidelines recommended and promoted their consumption as healthier options than animal-derived products (Food And Agriculture Organization of the United NationsF, 2016; World Health Organization, 2021). On the other hand, the overconsumption of animal products was reported to be related to increased incidences of cardiovascular diseases, diabetes, and hypertension (Hölker et al., 2019; O'Connor et al., 2017).

### 2.2 New generation in plant-based alternatives

Innovation in formulation/processing were the key drivers to create the new generation of plant-based foods with similar features (flavour, taste, and texture) to the conventional animal-based product. For instance, several companies have invested in flavouring and colouring solutions to be included in plant-based foods to mimic the authentic taste and appearance of beef and chicken meat. Masking agents have also played a relevant role in mitigating plant-based off-flavours and astringency. Binding and thickening agents, such as hydrocolloids, are equally important in creating a texture like the conventional products. Furthermore, the advances in processing technologies through the improvement of traditional methods, such as extrusion, and developing new tools, such as electrospinning and 3D printing, aimed to create a meat-like experience through plant protein texturization (from globular to fibrous structure) (Boukid, 2021). Beyond alternative products to meat, egg, seafood, and milk, traditionally consumed plant-based products such as bread, soups, cereals snacks and bars are being upgraded owing to the new plant-based ingredients. For instance, textured vegetable proteins (a key ingredient in meat alternatives) are being increasingly used in snacks (for crunchiness and to boost protein) and soups (for thickening and binding) (Baune et al., 2022).

### 2.3 Safety and nutrition are future key points

#### 2.3.1 Toxins and allergens

Plant-based foods may contain genetically modified organisms (GMO), allergens, anti-nutrients and processing contaminants (Hadi and Brightwell, 2021). The safety of GMO is still a debatable subject due to controversial evidence about their potential health and environmental risks. Available scientific evidence shows an unlikely risk of allergenicity, cross reactivity or toxicity in GM crops and derived ingredients (Reyes et al., 2021). Others suggest that commercialized GM soy and recombinant soy leghemoglobin might be related to new toxins and allergens due to undesirable mutations (Bøhn and Millstone, 2019; Shen et al., 2022). Their commercialization is still banned in some countries due to insufficient information about their safety over time. Some consumers may have allergies or intolerances to specific plant proteins used in plant-based foods, e.g., wheat. Processing can reduce but does not completely abolish the allergenic potential and thus allergens need to be declared on the label. Anti-nutritional components present in plants can cause health issues if they exceed a critical amount, but modern breeding and processing made great improvement in lowering their contents in plant-based ingredients and foods. For example, research has shown that fermentation or enzyme treatment can reduce the content of phytic acid in processed plant-based foods (Kaleda et al., 2020; Xing et al., 2020). Future research can explore further methods to improve the nutritional profile and overall healthiness of plant-based foods.

#### 2.3.2 Ultra-processed nature and its impact on health

According to NOVA classification based on the degree of processing of foods, most plant-based ingredients (e.g., protein isolate, protein concentrates, and textured vegetable proteins) and plant-based alternatives are considered ultra-processed (group 4). Additionally, several additives are being used in formulating plant-based products and this is alarming for some consumers (McClements and Grossmann, 2021; Messina et al., 2022; Ohlau et al., 2022). Although some ultra-processed foods are associated with health concerns such as cancer, obesity and cardiovascular diseases (Julia et al., 2018; Satija et al., 2017), it is not a given that less processed foods are healthier (Bryant, 2022). Moreover, limited research has focused on the impact of plant-based foods on human health at short and long terms. Therefore, the health benefits of plant-based foods cannot be condemned only by the degree of processing but it must also consider product formulations, frequency of consumption, and the dietary pattern of the individual (Crimarco et al., 2020; van Vliet et al., 2020). Limiting quality evaluation of plant-based foods to NOVA is not enough to determine the healthiness of these foods. Probably, combining NOVA and Nutri-score would give a better understanding about the quality of the foods to consumer, using these indexes as criteria for purchase decision.

#### 2.3.3 Unbalanced nutritional composition

Even though plant sources are rich in health-beneficial compounds (e.g., protein, carbohydrates, fiber and minerals) unbalanced plant-based diets can result in malnutrition or under nutrition contrarily to adequate diets (Sakkas et al., 2020; Sebastiani et al., 2019). This can be attributed in part to the protein composition of some plant sources lacking essential amino acids, and this can result in protein digestibility-corrected amino acid score (PDCAAS) lower than animal protein sources. Blending complementary plant sources would enable a complete essential amino acids profile. Plant-based sources are also lower in essential elements like vitamin B12 and iron, commonly available in animal products. Biofortification in vitamins and minerals is therefore required to have a well-balanced vegan diet. Some plant-based foods were reported to have lower protein, higher carbohydrates, sugar, and salt contents compared to conventional products (Boukid and Castellari, 2021; Pointke and Pawelzik, 2022). However, other products showed low saturated fatty acids and cholesterol content and high fiber content (Boukid and Castellari, 2021; Guess et al., 2022; Salomé et al., 2021). This is due to the lack of nutritional standardization versus the high number of products formulated differently and currently available in the global market. This suggests the need to clear regulations of the nutritional composition to alternative products. Vegan trend is also progressing in infant foods (e.g., cholesterol free), yet in this case consuming exclusively plant-based foods could result in low levels of micronutrients such as calcium, iron, and vitamin B12, essential for the growth of infants. Thus, complete and informative nutritional labelling is necessary to make a conscious decision (Boukid and Castellari, 2021; Ohlau et al., 2022).

### 2.4 Sourcing new plant-based ingredients

Although a wide portfolio of plant-based ingredients is currently available, the production of plant-based crops is unstable, and the new crops being explored to keep up with market demand have a low productivity. Plant crops are extremely vulnerable to climate change. Higher temperatures negatively impact crop productivity and encourage undesirable plants and pest proliferation. Changes in precipitation patterns might increase the possibility of crop failures and decrease crop productivity. A growing population is an important driver of increased food (plant and animal) demand, which is closely related to land, water, and energy resources.

Furthermore, recent events such as the COVID-19 outbreak and the Russian invasion of Ukraine are serious matters that impact food security. The COVID-19 outbreak directly impacted the animal-based market and contributed to the raise of the demand of plant-based food as healthier options (Di Renzo et al., 2020; Rzymski et al., 2021). In 2022, the Ukraine crisis impacted the market prices of cereals and legumes since Russia and Ukraine are major producers of cereals (Jagtap et al., 2022). Prices increased in Europe and to provide for the demand for cereals, high amounts were imported from South America (mainly Brazil) and North America. These events make it necessary to explore new plant sources, largely available, such as the biomass made up of grass or vegetable waste deriving from the processing agro-industrial crops (sugar beet, cassava, olive, etc.). Aquatic biomass, e.g., micro and macro algae, and aquatic plants such as duckweed, are another promising alternative as a food source, with good protein conversion efficiency (Tamayo Tenorio et al., 2018). Also, *Moringa oleifera* is a valuable protein source, and an alternative to pulses. However, comprehensive information should always accompany the most innovative products to reduce possible phenomena of food neophobia (Pasqualone, 2022).

### 2.5 Sustainability

Plant crops are generally sustainable crops with lower ecological footprint compared to animal production (Alcorta et al., 2021). For instance, the recovery of proteins as side streams from starch or oil extraction contributes to a circular economy. Nevertheless, wet extraction of proteins uses high amounts of water and energy and generates a lot of waste. Alternatively, the use of dry fractionation or mild extraction for protein concentrates is much more sustainable (de Angelis et al., 2020). Overall, existing scientific evidence about the environmental impact of plant-based ingredients and foods strongly supports plant-based products as more sustainable than animal products but cannot be used as a solid base to draw widely generalizable conclusions, since plant-based foods processing differs and thus each product is a special case. This can be taken into consideration to reduce the impact on the environment and find sustainable plant-based approaches to fight hunger and food insecurity.

## 3 Digitalisation in agriculture and the food industry

**Fig. 3:**
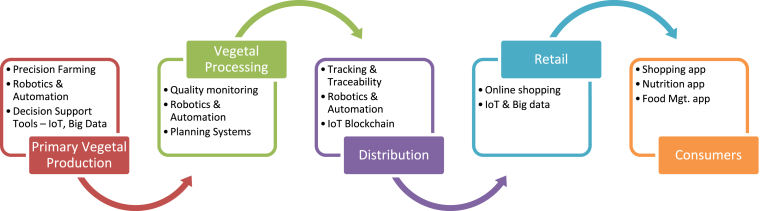
Digitalisation in the vegetal supply chain.

Digitalisation is continuously evolving with the aim to enhance productivity, reduce food safety risk, and improve the overall sustainability of the vegetal supply chain (Grote et al., 2021). Many Industry 4.0 technologies such as AI, IoT, BD, blockchain, robotics, and smart sensors are being employed by the whole vegetal supply chain from farm to fork (Hassoun et al., 2023; Javaid et al., 2022; Martindale et al., 2022). Fig. 3 shows the digitalisation in the vegetal supply chain starting at the primary production, i.e., at the farm level and concluding at the consumer level. It also shows various digital technologies being implemented by various actors all along the vegetal supply chain, as described below.

### 3.1 Primary vegetal production

At the primary production level, digitalisation is deployed to reduce the use of pesticides, insecticides, fertilizers and to ensure appropriate irrigation (Talaviya et al., 2020). IoT and BD combined with weather data are extensively used for accurate farm management and the development of better decision support tools (Jagtap et al., 2019; Wolfert et al., 2017). The use of robotics and automation for harvesting is on the rise (Duong et al., 2020). We have also seen the use of agricultural drones for crop mapping, pest management and insecticide spraying (Filho et al., 2020).

### 3.2 Vegetal processing

Extensive vegetal data is being collected at the processing level in order to establish key quality parameters such as ripeness level, moisture, sugar content, size, as well as foreign matter (Chen et al., 2013). This has led to improved transparency and more control over food quality and safety issues (Jagtap and Duong, 2019). In addition, robots and automation are used increasingly for cleaning, grading, sorting, and packing purposes (Duong et al., 2020).

### 3.3 Distribution

At the distribution level, IoT-based blockchain technology is increasingly used for fruits and vegetables for real-time tracking and tracing purposes (Trollman et al., 2022). In addition, IoT is being used for better management of stocks in warehouses (Jagtap et al., 2021a), and for road traffic congestions, BD is being utilised (Jagtap et al., 2021b; Zhang, 2013) for enhancing vehicle performance through predictive maintenance, and blockchain for encrypting important contract documentations and eliminating intermediaries. Some examples include Twiga Foods, a blockchain-powered supply chain platform to connect primary producers with vendors to allow food to be sourced and distributed across Kenya (Nair and Landani, 2020). Similarly, the Fleet app uses GPS to track the fleet and Asset Tracker allows remote tracking, monitoring, and management of shipments (Jagtap et al., 2021; Zhang, 2013).

### 3.4 Retail

Online shopping has increased exponentially around the globe after the COVID-19, and many retailers have adopted advanced use of digital solutions. For example, Ocado, an online grocery retailer based in the UK, uses robots, IoT sensors and vision systems to pick customer orders in their large warehouses, making them faster, and more reliable and economical than traditional warehouses (Mahroof, 2019). The digitalisation trend in the German food online grocery retail has been studied during the COVID-19 pandemic (Dannenberg et al., 2020). Although the growth rates in the online food trade have experienced a strong upswing since the start of the pandemic, the spatial diffusion of online food grocery was found to be still limited. A recent study investigated the potential of applying advanced data-driven strategies, such as BD and real-time IoT sensors to reduce food waste at the retail level (Kayikci et al., 2022).

### 3.5 Consumers

Many applications are developed to support consumers with future shopping, personalised nutrition, and food management (Jagtap and Rahimifard, 2019). Some apps track future orders of consumers, the nutrition and diet requirements of each consumer, and suggest recipes before food expires. For example, mobile food ordering apps are increasingly used to provide consumers with more comprehensive, up-to-date, and accurate information about the restaurants and the menu options (Alalwan, 2020). Nowadays, applications are being developed and integrated into smartphones to guide consumers in their choice of healthy and fresh foods. For instance, a smartphone-integrated colorimetric sensor, based on the qualification of total volatile basic nitrogen was developed and successfully used to monitor fish freshness in a rapid and non-destructive manner (Zhang et al., 2021). Other smartphone-based technologies can be used to help consumers make better-informed decisions about the food they order, and remind them of the food expiration date, in addition to other detection, analysis, diagnosis and monitoring applications (Hussain, 2021).

## 4 Interconnection between vegetal and digital trends

This section discusses how digitalisation in the supply chain can support the vegetal sector and therefore a shift to more plant-based diets.

### 4.1 Internet of Things (IoT)

IoT is one of the concepts framed within Industry 4.0 with more potential to shape businesses in the short to medium term. The IoT market value was USD385 billion in 2021, with predictions to reach more than USD2, 400 billion by 2029 (Fortune Business Insights, 2022). IoT is a network of connected devices that collect and share data with other devices and systems. This allows easy access to live data as well as its storage and distribution to stakeholders and decision makers. Consequently, processes and products can be monitored in real-time, and thus accelerating the move toward precision agriculture technologies and smart farming practices (Boursianis et al., 2022; Javaid et al., 2022).

IoT is slowly being embraced by large food businesses. For instance, IoT-based technologies such as remote monitoring systems, decision support tools, automated irrigation systems, frost protection systems, and fertilization systems support agricultural operations (Gómez-Chabla et al., 2019). Most IoT applications in the food industry focus on the control of traceability, temperature, humidity, colour, and enhancement of sustainability performance (Endres et al., 2022). Such applications are particularly important in the vegetable supply chain, especially at the agricultural stage, where accurate control of indicators is often key to enhancing crop productivity. For instance, operational parameters such as pesticide and water use have been optimised by using IoT systems (Moysiadis et al., 2021). Other parameters that have been controlled via IoT include soil nutritional content, humidity, temperature, and plant physiology, such as the vegetative index, nutritional requirements, electrical conductivity, and magnetic susceptibility (Maraveas and Bartzanas, 2021). All this information allows more precise crop monitoring.

The data collected by IoT can then be used to react more quickly to disruptions and to make more informed decisions, with the final aim of improving the economic performance of agricultural businesses. However, a common positive side effect is the combined enhancement of the environmental performance of both the product and the business. Gautham et al. (2022) combined machine learning and IoT in a humidification control system that reduces post-harvest losses of vegetables by automatically controlling the storage temperature and humidity. This reduces economic costs and environmental impacts of agricultural businesses.

Karmakar et al. (2022) developed an IoT-based framework, namely I-Fresh, that determines the freshness of vegetables by sensing and analysing their colour. The information about the quality of the product, collected via IoT, can then be used to predict the fair price of a vegetable product (Wijekoon et al., 2021). This is particularly useful in the vegetable sector, where product quality significantly varies over seasons, planting locations or even in the same field. Furthermore, knowing the exact quality and composition of the product is also useful in detecting and rejecting non-conforming products, e.g., those that have spoiled or that present toxins or allergens.

IoT is already shaping the vegetable supply chain and is expected to continue to do so in the future. Chamara et al. (2022) identified the following areas where IoT can play an important role for vegetable production: integration of satellite-based internet connectivity to improve the IoT networks in non-connected farms, development of mobile IoT platforms (such as drones and autonomous vehicles) with continuous connectivity, and the use of edge-computing and machine learning to enhance the capability of the agricultural systems.

Innovation and advanced technologies are also being used to valorise new food sources, such as algae. For example, IoT was reported to help automate algal manufacturing technology and drive the establishment of an algal-based bioeconomy (Fabris et al., 2020). In conclusion, IoT can boost the digitalisation of the agricultural sector while also accelerating the shift towards more vegetable diets.

### 4.2 Artificial intelligence (AI)

Currently, an AI revolution is happening in almost all industries (including the agriculture and food industry) on a global scale. Two examples of the application of AI in the agri-food sector are discussed in this section, while many other applications can be found in other recent publications (Addanki et al., 2022; Ben Ayed and Hanana, 2021; Ramirez-Asis et al., 2022; Talaviya et al., 2020).

#### 4.2.1 Improving food with AI

In the food science and technology literature, “Rational Food Design” (RFD) aims to meet consumer needs by assembling food microstructures and matrices that deliver the target psychophysical, nutritional or health functionalities (Aguilera, 2022). RFD of food structures is expected to have key roles in providing flavourful plant-based diets and product personalization (Alcorta et al., 2021). AI will ideally support the rational design of plant-based products as information on ingredients, recipes, operational conditions, and sensorial responses. These databases will need to be mined for pattern identification and useful information relevant to product design supported by machine-learning algorithms that can then also propose viable design alternatives (Aguilera, 2022). Further up the supply chain, omics technologies integrated with AI approaches can be used to improve crops for functional foods and nutritional security (Nayak et al., 2021).

#### 4.2.2 Cellular agriculture and AI

Interest in microalgae and plant cells as sources of ingredients for the food industry continues to increase (Jönsson, 2020). Microalgal bioreactors are engineered ecosystems that cultivate algal biomass for alternative protein sources and algae-derived products. However, the scale-up of bioreactors is a significant barrier to industrial implementation. In addition, current bioreactors are prone to contamination, including harmful cyanobacterial species, which affect product quality (consumer acceptance).

Low-resolution Raman spectroscopy supported by machine learning has been shown to be effective at identifying the presence of microalgae in water samples, detecting cyanobacteria or algal cells at a concentration of 10^4^ cells/mL or lower using direct measurement with no sample preparation (Adejimi et al., 2022). However, machine learning models have yet to achieve their full predictive potential in uncovering the relations between inputs and outputs. Barriers to building machine learning models include a lack of data for omics technologies and the need for explainable artificial intelligence to make the results of AI methods understandable to humans (Helmy et al., 2020). The next generation of cellular agriculture technologies is expected to employ predictive biology, supported by machine learning-guided systems biology and bioinformatics, for pathway discovery, enzyme identification, protein modelling, safety investigation and taste/texture modelling (Helmy et al., 2020; Smith et al., 2022).

### 4.3 Big data

Big Data (BD) refers to large, fast and complex data that cannot be processed and managed by classical and traditional techniques. Big Data Analytics (BDA) is the process of using sophisticated analytics on BD (Ciccullo et al., 2022). The most current or potential applications of BD in vegetables, fruits and other plant-based sectors include optimal planting of fruit trees using data extracted from satellite and unmanned aerial vehicle imagery (Saldana Ochoa and Guo, 2019), characterisation of size and shape phenotypes of horticultural crops using high throughput imagery (Haque et al., 2021), improvement of controlled environment agriculture, such as soilless hydroponics and others for vegetable and fruit farming (Ragaveena et al., 2021), and mitigating post-harvest losses and managing fruit and vegetable quality through machine learning (Singh et al., 2022).

There are also some applications of BD focused on driving demand and meeting consumer needs for plant-based foods, such as developing new smart fruit marketing models in e-commerce (Ma and Zhang, 2022), satisfying date consumers through an automatic image classification system based on 5G technology and deep learning (Hossain et al., 2018), and generating a healthy food recommendation for the end-user in a nutrition-based vegetable system (Ludena and Ahrary, 2016). There is another group of BD applications that cut across the entire agri-food chain, including certifying the quality of olive oil, one of the star products of the Mediterranean diet, using DNA traceability techniques in combination with 4.0 technologies including BD (Ben Ayed et al., 2022), improving dynamic risk management associated to food-borne pathogens leafy vegetables (Donaghy et al., 2021), improving food quality and safety inspection (including fruit) through deep learning (Chen and Yu, 2022; Zhou et al., 2019), improving the taste of vegetables by integrating metabolic profiling with other omics methodologies derived from BD (Zhu et al., 2019), and assessing the quality of fruits and vegetables using techniques such as computer vision, image processing and hyperspectral imaging (Bandal and Thirugnanam, 2016).

### 4.4 Other digital technologies

The Industry 4.0 and digitalisation is far from being limited to IoT, AI, and BD. The agri-food sector is increasingly embracing other advanced technologies, such as 3D printing, robotics, and blockchain. The need for these advanced technologies in agriculture and the food industry has been exacerbated by the COVID-19 outbreak (Galanakis et al., 2021; Rowan and Galanakis, 2020; Sarfraz et al., 2021; Wang et al., 2022).

3D printing (or additive manufacturing) can play significant roles in improving many aspects of agriculture and food sectors by developing customized nutrition plans and specifically tailoring food properties for individuals, such as elderly populations and sportspersons (Hassoun et al., 2022e; Zhang et al., 2022). For example 3D printing can be used for printing foods that resemble plant tissues or producing plant-based meats or seafood analogues (Baiano, 2020; Kazir and Livney, 2021). This technique can be even used to develop new products from food waste and by-products, thus boosting food sustainability and circular economy (Baiano, 2020; Jagadiswaran et al., 2021). For instance, a recent study highlighted the possibility of preparing noodles from potato peel waste using 3D food printing (Muthurajan et al., 2021).

Automation and robotization are other aspects of digital technology and play significant roles in agriculture to achieve smart farming/precision agriculture and in the food industry to accelerate the move toward smart factories. Nowadays, advanced robots and drones are equipped with smart sensors and can cooperate with humans to perform many tasks in the production field, such as crop production (such as seeding, planting, weeding, picking, handling, and harvesting) or during the later stage of the food manufacturing chain, e.g., packaging, cutting, slicing, and packaging (Botta et al., 2022; Hesti, 2021).

Blockchains are becoming increasingly used in many fields, allowing data to be shared between different actors in value chains in a reliable and transparent manner. Blockchains, especially when used in combination with other Industry 4.0 technologies, such as IoT, have the potential to address many challenges and solve complex problems in the food supply chain (Vistro et al., 2021). As an example, the grape wine supply chain was studied to show how cost-effective blockchain architecture could ensure food quality, food safety, and improve the agri-food supply chain efficiency and quality management (Saurabh and Dey, 2021). A recent publication demonstrated that the use of blockchain in combination with IoT can reduce costs and improve traceability in the fresh fruit supply chain (Zhang et al., 2022).

## 5 Conclusion

In recent years, agriculture and the food industry have experienced significant disruptions due to pandemic outbreaks, large-scale geopolitical conflicts, and climate change, among others. Agri-food systems are undergoing a digital transformation at different stages along the food supply chain, from farmers to consumers, covering the food production (precision agriculture/smart farming) and food manufacturing industries (smart food factories). This work briefly discussed recent developments in digitalisation and the newest innovations in plant-based foods and the interconnection between vegetal and digital trends.

Currently, the market of vegetal products is gaining momentum with the growing demand for plant-based food products that are increasingly proposed as sustainable alternatives to conventional meat, seafood, egg, and dairy. Numerous obstacles, mainly related to consumers’ acceptance of sensory properties and nutritional quality, not to mention a certain psychological reluctance towards new sources (food neophobia), are still hindering their wider commercialization, but these issues could be mitigated by comprehensive information on the advantages and by properly incorporating innovation and emerging technologies. Indeed, Industry 4.0 technologies, including AI, BD, IoT, blockchain, robotics and smart sensors have been increasingly applied to solve many challenges in the agri-food sector. Increasing evidence shows that digital technologies and other technological advances could be leveraged to accelerate the shift to vegetal diets, thus contributing to the transition to more sustainable food systems. The whole journey of plant-based food starting from the primary vegetal production, to processing, distribution, retail, and finally to the consumer, could be assisted through digital solutions that could increase food nutritional quality, safety, and transparency.

For example, IoT-based technologies are being used at various stages along the vegetable supply chain, to help achieve accurate control and monitoring of numerous agricultural operations, enhancing crop productivity. Implementation of AI-based solutions can provide many opportunities at the agricultural stage to test soil, improve crop yield, detect ripe products automatically, predict crop diseases, etc. or at later manufacturing stages to develop new recipes for plant-based products. Other digital and Industry 4.0 technologies are revolutionising the agri-food sector ad taking it to the next generations, enabling smart food production and manufacturing.

However, investment and knowledge and skills required to ensure the successful implementation of new technologies, in addition to technophobia and resistance to change (*silo* mentality) could be highlighted as significant barriers facing the digital revolution. More multidisciplinary research is encouraged to overcome current limitations, build a balanced sustainable future food, and set food systems on a better course.

## CRediT authorship contribution statement

**Abdo Hassoun:** Conceptualization, Methodology, Data curation, Writing – review & editing. **Fatma Boukid:** Writing – original draft, Writing – review & editing. **Antonella Pasqualone:** Writing – original draft, Writing – review & editing. **Christopher J. Bryant:** Writing – original draft, Writing – review & editing. **Guillermo García García:** Writing – original draft, Writing – review & editing. **Carlos Parra-López:** Writing – original draft, Writing – review & editing. **Sandeep Jagtap:** Writing – original draft, Writing – review & editing. **Hana Trollman:** Writing – original draft, Writing – review & editing. **Janna Cropotova:** Writing – original draft, Writing – review & editing, Funding acquisition. **Francisco J. Barba:** Writing – original draft, Writing – review & editing.

## Declaration of competing interest

The authors declare that they have no known competing financial interests or personal relationships that could have appeared to influence the work reported in this paper.

## Data Availability

No data was used for the research described in the article.
